# Editorial: New work demands and managing employee well-being in the post-pandemic world

**DOI:** 10.3389/fpsyg.2024.1392687

**Published:** 2024-06-12

**Authors:** Zahid Riaz, Živile Stankeviciute, Florina Pinzaru

**Affiliations:** ^1^Lahore School of Economics, Lahore, Pakistan; ^2^Kaunas University of Technology, Kaunas, Lithuania; ^3^National University of Political Studies and Public Administration, Bucharest, Romania

**Keywords:** new work demands, employee wellbeing, work engagement (WE), algorithmic management of work, job crafting, remote work, person-organization fit (P-O fit)

From remote work to increased reliance on technology, employees have had to adapt to new practices and navigate the new work demands since 2020. The post-pandemic era witnessed Great Resignation, Quiet Quitting, and Loud Quitting, and thus, it has become necessary to examine employee happiness and wellbeing more closely (Formica and Sfodera, 2022; Moon et al., 2023; Larson, 2024). Employee wellbeing has become one of the most critical and pressing concerns (Zicari and Gamble, 2023). Employers and organizations have realized the importance of prioritizing their employees' physical, safety, and mental health (Kane et al., 2021). This shift toward a holistic employee wellbeing model is very crucial for the long-term success of organizations (Cao et al., 2022). A 2023 survey of Gallup into employee wellbeing discovers that having a job that employees hate is worse than being unemployed. Thereby, these negative emotions impact employee engagement and cause decreased productivity in the workforce. These productivity losses are estimated to account for about US $8.8 trillion, which is 9% of the global GDP (Gallup, 2023). Similarly, McKinsey Health Institute found that one out of four employees around the globe experiences burnout due to a toxic workplace (McKinsey, 2022). In a 2023 survey, it was found that employees who had positive work experiences were more innovative and better performers in their jobs (McKinsey, 2023). Such empirical evidence underscores the increasing recognition of the importance of employee wellbeing in the post-pandemic world, driven by a growing demand for organizations to contribute positively to society.

This Research Topic has explored the above-stated issues by presenting six high-quality manuscripts that examine how the new work demands influence employee wellbeing in the post-pandemic world. Our Research Topic differs from other new work demands-related literature by including papers attempting to uncover the underlying factors contributing to employee wellbeing, such as job crafting, autonomy, person-organization fit, organizational learning climate, organizational support, and individual, structural, and behavioral factors. These aspects have been examined across both private and public organizations and the selected articles have used both qualitative and quantitative research strategies. A summary of published articles on this Research Topic is provided below.

In today's rapidly changing work environment, self-managing organizations have gained attention due to their potential to promote various positive outcomes for the wellbeing of employees. The ability to make decisions autonomously, engage in job crafting behaviors, and effectively manage errors can significantly impact an individual's work engagement and job satisfaction. Understanding the complex interplay between perceived and ideal autonomy, job crafting, and error management orientation is crucial for creating a supportive and empowering work environment. In this context, Doblinger's paper delves deeper into the concept of autonomy, job crafting, and their relationships with the overall satisfaction and engagement of employees through a cross-sectional research design. The research findings highlight how aligning perceived autonomy with individual preferences can foster optimal work engagement and job satisfaction. Also, these results demonstrate the importance of individual autonomy for managers in self-managing organizations. The study findings imply reducing the error strain while enabling learning from errors and accepting the possibility of errors. These managerial interventions can improve job crafting and work engagement in the context of self-managing organizations.

Felix et al. explore in their study autonomy and security among gig workers in Brazil that can influence employee wellbeing by interviewing 57 workers in the gig economy during 2021–22. The research findings demonstrate that preference alignment only sometimes improves wellbeing. Both workers and organizations desire autonomy over security, but this can lead to potential abuse, such as unsustainable workloads, which can harm employee wellbeing. This study emphasizes that managers should take a balanced and integrated approach while evaluating the impact of algorithmic management on employee wellbeing. Thus, managers can move beyond the binary categorization of algorithmic management and acknowledge the complex interplay of preferences and their implications.

In a study of remote workers in the public services sector of Germany, Seinsche et al. investigate job demands and resources, as well as workers' perceptions regarding job satisfaction and productivity. The data was collected through the semi-structured telephonic interviews. Researchers found that employees new to working from home developed personal crafting strategies to adapt to their flexible working environment. These workers could effectively manage challenging job demands by optimizing their work environment. Moreover, employees utilized these strategies to optimize their social and structural resources. The time-spatial demands fit is closely linked to the use of job resources and job demands optimization, and it also combines work resources and demands to create an optimal work environment, enhancing employee productivity and satisfaction.

Vuuren et al. investigated how organizational learning climate, career commitment, and age are related to employees' self-perceived employability, vitality, and workability. In so doing, this study adopted a person-environment fit perspective. They surveyed 211 members of the support staff of a Dutch university. The study confirmed the importance of considering age and adopting a person-environment fit perspective on sustainable employability. Also, these findings imply that managers should create a work environment that supports learning for all employees, especially older workers. This is important because older employees may face challenges maintaining their ability to work sustainably due to age-related biases.

In the post-pandemic times, the career success models do not adhere to the conventional career trajectory. Hildred et al. examine the relationship between individual, structural, and behavioral factors and the objective and subjective career success of hybrid workers in Europe. This study extends the work of Spurk et al. (2019) regarding objective and subjective career success. The research findings underline the importance of individual, structural, and behavioral factors in shaping the career trajectories of remote and hybrid workers. Indeed, the “away from the office” phenomenon highlights managers' need for more pragmatic support and grooming initiatives for career advancement. For instance, managers can provide specific training and development programs. These organizational managers can empower their employees to steer their career progression in non-traditional work arrangements.

Bai et al. investigate the effect of psychological capital on the psychological wellbeing of tour guides in China. In so doing, the mediating role of work-family conflict, family-work conflict, work-family facilitation, and family-work facilitation is examined. Also, this research explores the moderating effect of perceived organizational support. The data was collected from 276 tour guides in 2021. According to the research findings, psychological capital reduces work-family conflict and improves work-family facilitation. Hence, it has a beneficial effect on the psychological wellbeing of tour guides. The moderating role of perceived organizational support underlines the importance of supportive organizational environments to influence the impact of work-family dynamics on psychological wellbeing. These findings imply that managers can develop strategies and interventions that can nourish the mental health and wellbeing of tour guides, especially during times of crisis.

These articles articulate the complexities of nuanced factors that can influence employee wellbeing in the post-pandemic world. This Research Topic implies that organizations should proactively ensure employee wellbeing through such interventions, which can be tailored to the unique challenges of diverse work arrangements. The post-pandemic organizational landscape commands vital shifts in the current workplace that have prompted a satisfactory examination of employee wellbeing and its influence on organizational success. Our Research Topic underscores the importance of prioritizing comprehensive strategies to support employee wellbeing in the post-pandemic world, enabling them to achieve optimal performance and satisfaction.

## Author contributions

ZR: Writing – original draft, Writing – review & editing. ŽS: Writing – review & editing. FP: Writing – review & editing.
